# Environmental impacts of food consumption by dogs and cats

**DOI:** 10.1371/journal.pone.0181301

**Published:** 2017-08-02

**Authors:** Gregory S. Okin

**Affiliations:** Department of Geography, University of California Los Angeles, Los Angeles, California, United States of America; University of Sydney, AUSTRALIA

## Abstract

In the US, there are more than 163 million dogs and cats that consume, as a significant portion of their diet, animal products and therefore potentially constitute a considerable dietary footprint. Here, the energy and animal-derived product consumption of these pets in the US is evaluated for the first time, as are the environmental impacts from the animal products fed to them, including feces production. In the US, dogs and cats consume about 19% ± 2% of the amount of dietary energy that humans do (203 ± 15 PJ yr^-1^ vs. 1051 ± 9 PJ yr^-1^) and 33% ± 9% of the animal-derived energy (67 ± 17 PJ yr^-1^ vs. 206 ± 2 PJ yr^-1^). They produce about 30% ± 13%, by mass, as much feces as Americans (5.1 ± Tg yr^-1^ vs. 17.2 Tg yr^-1^), and through their diet, constitute about 25–30% of the environmental impacts from animal production in terms of the use of land, water, fossil fuel, phosphate, and biocides. Dog and cat animal product consumption is responsible for release of up to 64 ± 16 million tons CO_2_-equivalent methane and nitrous oxide, two powerful greenhouse gasses (GHGs). Americans are the largest pet owners in the world, but the tradition of pet ownership in the US has considerable costs. As pet ownership increases in some developing countries, especially China, and trends continue in pet food toward higher content and quality of meat, globally, pet ownership will compound the environmental impacts of human dietary choices. Reducing the rate of dog and cat ownership, perhaps in favor of other pets that offer similar health and emotional benefits would considerably reduce these impacts. Simultaneous industry-wide efforts to reduce overfeeding, reduce waste, and find alternative sources of protein will also reduce these impacts.

## Introduction

Dietary choices have considerable impacts on environmental sustainability [1]. Compared to a plant-based diet, a meat-based diet requires more energy, land, and water and has greater environmental consequences in terms of erosion, pesticides, and waste. With over 7 billion human beings on the planet, increasing attention has been paid to the environmental effects of peoples’ diets, with some predicting a 100–110% increase in demand for agricultural production by 2050, which could require ~ 1 billion hectares to be cleared globally for agriculture [2]. Meat consumption, already high in developed nations, is increasing in developing nations as the standard of living increases [1, 3–5]. In addition to requiring greater land compared to plant crops to produce equivalent protein energy, and contributing to soil erosion, animal production has considerably greater impacts on water use, fossil fuel use, greenhouse gas emission, fertilizer use, and pesticide use [2–7]. Despite the fact that more than 60% of US households have pets [8], these consumers of agricultural products are rarely included in calculations of the environmental impact of dietary choices.

Given the significant environmental impact of meat production, the contributions of our omnivorous and carnivorous pets deserve special attention. The US has the largest population of pet dogs and cats globally, with an estimated 77.8 million dogs and 85.6 million cats in 2015 [8]. The consequences of these animals on wildlife and water quality have been investigated, with studies showing considerable impacts on carbon usage [9, 10], water quality [11–14], disease [15–18] and wildlife [19–21]

Here, the contribution of dogs and cats to total US energy and meat consumption and the environmental impact of that meat consumption, including the production of feces, is considered. The goal of the study is to understand the scale of these animals' dietary needs in relation to those of Americans. The number of dog- and cat-owning households is increasing in the US [8], and at the same time there is an increasing trend in the “humanization” of pets and pet products [22, 23]. As a possible consequence, there is a trend toward increasing meat quantity and quality in pet foods, which results in further increases in consumption of animal products by pets. There is evidence that this trend may continue as younger people are more likely to purchase premium pet food that includes more desirable cuts of meat [24]. Globally, the increasing pet ownership in developing countries [25, 26] may serve to increase the potential environmental impacts of pet dogs and cats.

## Methods and results

### Total energy consumed

Energy consumption was calculated as:

$$E_{Total}^{a}=E_{Indvidual}^{a}N$$ (1)

where $E_{Total}^{a}$ is the total energy consumed annually, $E_{Indvidual}^{a}$ is the per-capital annual consumption, and *N* is the number of individuals. $E_{Indvidual}^{a}$ was calculated separately for humans, dogs, and cats.

The Census Bureau estimates that the total population of the US was 321 million in 2015, with roughly equal proportions of men and women [27] (Table 1). The USDA Agricultural Research Service estimates that on average, US males (age 2+) consume 10,330 ± 91 kJ d^-1^ (2,469 ± 81 kcal d^-1^) and US females (age 2+) consume 7,607 ± 64 kJ d^-1^ (1,817 ± 15 kcal d^-1^). Therefore, the average daily energy consumption for both males and females is 8,966 ± 155 kJ d^-1^ (2,143 ± 37 kcal d^-1^) [28]. Using Eq 1, these estimates result in a total human energy intake of 1,051 ± 9 PJ y^-1^.

**Table 1:** Population and energy requirements of US people, dogs, and cats.

	Number (millions)	Energy Usage (KJ day^-1^ cap^-1^)	Annual Energy Use (PJ/yr)
Men	160.5	10330 ± 91	605 ± 5
Women	160.5	7602 ± 64	445 ± 4
	Men + Women		1051 ± 9
Dogs	77.8	5594 ± 443	159 ± 13
Cats	85.6	1426 ± 79	45 ± 2
	Dogs + Cats		203 ± 15
Percent of humans' energy used by dogs and cats			19% + 2%
Number (in millions) of Americans that eat as many calories as US dogs and cats			62 ± 5

The American Pet Products Association (APPA) estimates that there were 77.8 million dog and 85.6 million cats owned as pets in the United States in 2015 (Table 1) [8]. Dogs’ energy requirements are taken as ~544 kJ (kg BW)^-0.75^ d^-1^ [29]. Dogs’ body weight (BW) varies greatly by breed. To estimate the average BW of dogs, the average weight of the American Kennel Club (AKC)’s list of the 10 most popular dog breeds in the US was used [30]. Average breed weights were taken either from the AKC or other sources [31]. This resulted in an average US dog BW of 22 kg. The standard deviation of the average breed weights represents the variability among breeds, rather than uncertainty in the average dog weight and is therefore inappropriate for the uncertainty analysis done here. To estimate the uncertainty in the average dog weight, data from Meyer et al. [32] were taken for 10 breeds of different sizes. For each breed, Meyer et al. [32] reports the mass and standard deviation of the samples (n = 4 to 9). The standard deviation was regressed against the mass (r^2^ = 0.87) and standard deviation at 22 kg was estimated as 1.2 kg. Therefore, the estimated average US dog BW that will be used hereafter is 22 ± 1.2 kg giving an average energy requirement of 5,594 ± 443 kJ d^-1^ (1337 ± 106 kcal d^-1^). Multiplied by the estimated number of owned dogs in the US (Eq 1), this results in an estimate of 159 ± 13 PJ y^-1^ consumed by dogs [29].

Cats require ~544 kJ (kg BW)^-0.67^ d^-1^ energy [29]. The body weight of cats varies less than that of dogs, so the average and standard deviation of cat weight in Bermingham et al. [33] (4.2 ± 0.2 kg) were used to represent average cat weight, resulting in a total cat energy requirement of 1,426 ± 79 kJ d^-1^ (341 ± 19 kcal d^-1^). Multiplied by the estimated number of owned cats in the US (Eq 1), this results in an estimate of 45 ± 2.5 PJ y^-1^ consumed.

The proportion of the dietary energy in the US consumed by dogs and cats was calculated as the sum of the energy consumed by dogs and cats (203 ± 15 PJ y^-1^) divided by human energy intake (1051 ± 9 PJ y^-1^), with the result that dogs and cats consume about 19.4 ± 1.6% of the energy that humans in America do (Table 1).

### Energy from animal sources

For humans, the fraction of energy that is derived from animal sources, *F*_*A*_, can be calculated as:

$$F_{A}=\frac{E_{A,C}}{{\text{E}}_{C}^{a}},$$ (2)

where *E*_*A*,*C*_ is the energy consumed by humans from animal sources (subscript A). *E*_*A*,*C*_ can be calculated from data available from the U.S. Department of Agriculture (USDA) (Table 2): the total amount of red meat (including beef, veal, pork, and lamb), poultry (including chicken and turkey) and fish (including fish and shellfish) eaten by each Americans is 59.6 kg yr^-1^. Given the energy density of each food used by the USDA (Table 2), and with the conservative assumption that this meat provides the only animal-derived energy consumed by Americans, it is calculated that Americans consume 206 PJ yr^-1^ from animal sources, which constitutes 20% of their total energy intake.

**Table 2:** Per capita meat consumption in the US. Data from [28]. Uncertainty not available.

	Red Meat	Poultry	Fish	Total
Mass Consumed (kg yr^-1^ cap^-1^)	32.4	24.6	2.6	59.6
Energy Density (kJ g-1) ^a^	12.2	9.5	5.0	10.8 ^b^
Energy Consumed (MJ yr^-1^ cap^-1^)	393	235	13	641
Total calories from meat consumed per year by humans (PJ yr^-1^)				206
Percent of Americans' calories from meat				20% ^c^

^a^ Values used by the USDA in their calculations

^b^ Average of energy densities weighted by consumption.

^c^ Americans' total energy consumption from Table 1.

For dogs and cats, direct data on consumption is not available and therefore *F*_*A*_ cannot be calculated directly using Eq 2. Instead, new calculations must be made based on available data: ingredient lists for dog and cat foods and the composition of these ingredients in terms of substrates which have well-known energy densities (i.e., Atwater factors for protein, carbohydrate, and fat).

To do this, the ingredient lists for individual pet foods were used. Individual ingredients were considered in terms of the content of energy-providing substrates, protein, fat, and carbohydrate and non-energy providing components like water, ash, and fiber. Compositional data analysis is required for these calculations because the substrate components must sum to unity [34]. For a particular pet food, *m*, the center (analogous to the arithmetic mean) dry mass fraction of substrate *k* (protein, fat, carbohydrate, other), expressed as average grams of *k* per gram of *m*, was calculated as the closed geometric mean:

$$M_{k}^{m}={\left(\Pi_{i=1}^{5}M_{k}^{m,i}\right)}^{1/5},$$ (3)

where $M_{k}^{m,i}$ is the mass fraction of substrate *k* in one of the first five ingredients, *i*, in a particular food (i.e., grams of *k* per gram of *i*). For these calculations, the category ‘other’ was included to provide closure [35], that is, so that the fractions of all categories would sum to unity. $M_{k}^{m,i}$ was estimated for each ingredient by equating it with a general ingredient category for which substrate content is available (Table 3) [29, 36].

**Table 3:** Simplified ingredient list including dry matter content and content of protein, fat, and carbohydrates.

Ingredient	Animal-derived	% dry matter	Dry Weight Mass Percent			Weight Percent with Water Included		
			Protein	Fat	Carbs	Protein	Fat	Carbs
Animal-derived Broth (Dry)	*	96%	33	18	49	31	18	47
Animal-derived Broth (Liquid)	*	5%	33	18	49	2	1	2
Animal-derived Fat	*	99%	0	100	0	0	99	0
Animal-derived Protein	*	90%	100	0	0	90	0	0
Beef	*	30%	61	31	4	18	9	1
Beef (Dry)	*	90%	61	31	4	55	28	3
Beef and Bone Meal	*	65%	34	18	21	22	11	14
Bones	*	100%	8	3	10	8	3	10
Eggs	*	43%	22	14	46	9	6	20
Eggs (Dry)	*	90%	22	14	46	20	13	41
Fish	*	25%	22	14	46	6	4	12
Fish (Dry)	*	90%	68	8	3	61	8	3
Grain		90%	13	4	73	12	3	66
Lamb	*	40%	37	59	5	15	23	2
Lamb (Dry)	*	90%	61	31	4	55	28	3
Legumes		90%	36	4	49	32	3	44
Other Vegetable Source		10%	30	0	70	3	0	7
Plant-derived Broth		5%	33	18	49	2	1	2
Plant-derived Carbohydrate		0%	0	100	0	0	100	0
Plant-derived Fat		25%	22	14	46	6	4	12
Plant-derived Fiber		90%	18	1	81	16	1	72
Plant-derived Protein		90%	100	0	0	90	0	0
Pork	*	30%	72	15	7	22	5	2
Pork (Dry)	*	90%	72	15	7	65	14	7
Poultry	*	30%	61	31	4	18	9	1
Poultry (Dry)	*	90%	58	27	3	52	25	2
Poultry and Bone Meal	*	95%	33	15	6	31	14	6
Tuber		90%	9	1	80	8	1	72
Yeast		93%	48	2	32	45	2	30

Similarly, the average dry mass fraction of animal-derived substrate *k* for a particular food (i.e., average grams of animal-derived *k* per gram of *m*) was calculated as the closed geometric mean:

$$M_{A,k}^{m}={\left(\Pi_{i=1}^{5}M_{A,k}^{m,i}\right)}^{1/5}$$ (4)

where $M_{A,k}^{m,i}$ is the mass fraction of animal-derived substrate *k* in one of the first five ingredients, *i*, in a particular food (Table 3 asterisks indicate animal-derived). For these calculations, the same approach was used in calculation of $M_{k}^{m}$, except non-animal derived protein, fat, and carbohydrates were added to the ‘other’ category to maintain closure. $M_{k}^{m}$ and $M_{A,k}^{m}$ are (geometric) average mass fractions and therefore explicitly assume that the first five ingredients in a food are present in equal proportions and that they constitute nearly all of the mass of pet food *m*. This assumption is wrong, but conservative, as explained below. Uncertainty in $M_{A,k}^{m}$ was calculated as the variance across all *m* for each substrate *k* [35].

The fraction of energy derived from animal products in a food *m* (animal-derived joules per total joules) was calculated as:

$$F_{A}^{m}=\frac{\sum_{k}E_{k}M_{A,k}^{m}}{\sum_{k}E_{k}M_{k}^{m}},$$ (5)

where *E*_*k*_ is the energy density of the substrates (i.e., Atwater factors: *E*_*protein*_ = *E*_*carbohydrate*_ = 4J/g, *E*_*Fat*_ = 9 J/g [29]). *E*_*other*_ was set to zero in for both total and animal-derived calculations. In the former case, water, ash, and fiber, which provide no dietary energy, comprised the ‘other’ category. In the latter, ‘other’ contained water, ash, and fiber as well as non-animal derived protein, fat, and carbohydrates, on the logic that these do not provide animal-derived dietary energy. The total animal-derived energy was calculated as

$$F_{A}=\frac{E_{Dog}^{a}}{E_{Dog}^{a}+E_{Cat}^{a}}\left(P_{Dog,P}\frac{1}{M_{Dog,\;P}}\Sigma_{Dog,P}F_{A}^{m}+P_{Dog,N}\frac{1}{M_{Dog,\;N}}\Sigma_{Dog,N}F_{A}^{m}\right)\;+\frac{E_{Cat}^{a}}{E_{Dog}^{a}+E_{Cat}^{a}}\left(P_{Cat,P}\frac{1}{M_{Cat,\;P}}\Sigma_{Cat,P}F_{A}^{m}+P_{Cat,N}\frac{1}{M_{Cat,\;N}}\Sigma_{Cat,N}F_{A}^{m}\right),$$ (6)

which is the weighted average fraction of animal-derived energy in four categories: premium dog food (n = 102), market-leading dog food (n = 9), premium cat food (n = 163), and market-leading cat food (n = 9). $E_{Dog}^{a}$ is the annual total energy consumed by dogs and $E_{Cat}^{a}$ is the annual total energy consumed by cats (Table 1) *P*_*x*,*y*_ is the proportion of dog or cat owners and (*x* = *Dog* and *Cat*, respectively) who prefer premium or market-leading foods (*y* = *P* and *N*, respectively). Likewise, *M*_*x*,*y*_ is the number of foods considered here in each category. More premium foods were used in these calculations because there is more diversity in this market sector. For dry dog food, nine foods from just five manufacturers constitute 48% of the market [37]. For dry cat food, nine foods from just four manufacturers constitute 49% of the market share [38].

Dry foods were used for these calculations. For both dogs and cats, dry food sales dominate wet food sales (billions of US dollars in sales for various foods in 2012: 8.7 (dry dog food) vs 2.3 (wet dog food) [39], and 3.6 (dry cat food) vs. 2.4 (wet cat food) [40], and thus are more representative of the foods fed to cats, and especially, dogs. The dominance of dry food as the preferred form is especially true when the price per serving is taken into account. One market-leading wet cat food costs approximately $0.83 per serving while a dry food by the same manufacturer costs approximately $0.23 per serving. Using the this per-serving price ratio, dry cat food outsells wet cat food on a per-serving basis by a factor of about 3 to 1. Furthermore, dry food typically has lower animal content (as determined by the list of ingredients in descending order of mass contribution) than wet food. Thus, use of dry food for these calculations provides a conservative estimate of the greatest proportion of dog and cat food sales in the U.S.

USDA labeling rules require that pet food ingredients be labeled in descending order of weight contribution, as they do with foods intended for humans. Calculations were made on the assumptions that 1) each of the first five ingredients contributes, by mass, equally to the mass of the pet food and 2) collectively, these first five ingredients make up nearly all of the mass of the pet food (that is, there are no other ingredients that contribute substantially to the mass of the food). With regard to the former, for marketing purposes, animal-derived ingredients typically appear in in the top couple of places in the ingredient list. This is particularly true of premium foods, where 100% of both dog and cat foods examined here had animal-derived products as the first ingredient (Table 4). For all types of dry food examined here (market-leading v. premium dog and cat foods), animal-derived ingredients appear among the first two ingredients more commonly than among the third and fourth ingredients (Table 4). Thus, the calculations made here over-weight the later ingredients, which are less likely to be animal-derived, compared to the earlier ingredients, which are more likely to be animal derived. Although there is no way to know, in proprietary recipes, the exact proportions of ingredient, by weighting the first five ingredients equally, a minimum overall estimate of animal-derived energy in dog and cat food is produced.

**Table 4:** Frequency of an animal derived ingredient in one of the first two positions or one of the two following positions in the ingredient list of dry foods considered here.

Position	Dog		Cat	
	Market-leading	Premium	Market-leading	Premium
First or second	89%	100%	63%	100%
Third or fourth	56%	65%	38%	55%

With regard to the second assumption, that the first five ingredients make up nearly all of the mass of the pet food, ingredients appearing past the first five in the ingredient list are often nutrients (e.g., tocopherol) added in trace quantities. If ingredients past the fifth are not trace, then given the requirement that ingredients be listed in decreasing mass contribution, the sixth ingredient must contribute less than 16% of the mass of the food. In the case of seven substantive ingredients, the maximum fraction of the mass in the 6^th^ and 7^th^ places is 29%. Among the premium brands that were examined, the proportion of animal-derived product decreased as they occurred later in ingredient lists with only 21% of the sixth ingredients in dry dog food being animal-derived. Thus, even in the extreme case, a maximum of 3–6% (21% of 16% = 3.5%; 21% of 29% = 6%) of the animal-derived content may be missing in the foods examined here. Although the methodology use here cannot give exact amounts of animal-derived content from foods, the potential maximum exclusion of 3–6% of animal-derived products is sufficient to draw important conclusions about the amount of animal-derived energy consumed by dogs and cats.

The APPA’s annual pet-owners survey [8] provides data that can be used understand consumer preferences, thus providing information about ratio of premium vs. non-premium (market leading) foods consumed. Non-premium brands tend to have lower animal-derived content whereas premium brands tend to have higher animal-derived content. The premium brand category used here includes the 'premium' and 'gourmet' survey categories. For dogs of all sizes, the average percent of owners who usually feed these meat-rich dog foods is 38%. For cats, this number is 30% (Table 5).

**Table 5:** Values used in the calculation of the fraction (and total) of energy consumed by dogs and cats in the US.

	Dog		Cat	
Total Energy Consumed (PJ/yr)	159 ± 13		45 ± 2	
Fraction of Total Pet Energy Consumed	78%		22%	
Food Type	Premium	Market-leading	Premium	Market-leading
Fraction of calories from animal sources	47% ± 7%	25% ± 2%	47% ± 9%	24% ± 2%
Fraction of consumers preferring food type	38%	62%	30%	70%
Total fraction of calories derived from animals	34% ± 4%		31% ± 4%	
Energy-weighted fraction of consumed energy from animal sources	33% ± 6%			
Energy-weighted amount of consumed energy from animal sources (PJ yr^-1^)	67 ± 17			
Energy consumed by humans from animal sources (PJ yr^-1^) from Table 1	206 ± 2			
Proportion animal-derived energy: (Dog + Cat)/Human	33% ± 9%			
Proportion animal-derived energy: (Dog + Cat)/Total	25% ± 6%			

The final market-wide estimates of the fraction of energy in dog and cat foods that is animal-derived are 34% ± 4% and 31% ± 4%, respectively (Table 5). In total, Eq 6 yields an estimate that animal-derived energy constitutes 33% ± 6% of the diets of dogs and cats in the US. This is significantly higher than the fraction of humans’ dietary energy that is animal-derived (19%). Because dogs and cats consume, together, 203 ± 15 PJ/year, it is estimated that dogs and cats consume a minimum of 67 ± 17 PJ/year in animal-derived energy, which is 33% ± 9% of the animal-derived energy consumed by humans in the US or 25% ± 6% of the total.

An important caveat for the calculations of the relative consumption of pets and humans is that the sources of the data, and mode of calculation, are dramatically different. As a result, their ratios may be systematically biased. Nonetheless, the calculations of absolute amounts (e.g., PJ/yr) are informative, and the relative amounts still provide important insight into the magnitude of pets’ consumption.

### Plant-equivalent energy consumption

Calculating the dietary energy in animal-based diets compared to the equivalent plant energy required support animal production for those diets is an important way to contextualize different dietary choices (e.g., [41, 42]). Here, plant-equivalent energy calculations are used as a means to understand the scale of the impact of dogs' and cats' meat consumption in relation to the energy requirements of people. The plant-equivalent energy consumed by humans and animals can be calculated as:

$$E_{PE}=FCR_{E}F_{A}{\text{E}}_{C}^{a}+\left(1-F_{A}\right){\text{E}}_{C}^{a},$$ (7)

where *FCR*_*E*_ is the feed conversion ratio for meat on an energy (J/J) basis rather than the more common mass (g/g) basis. *FCR*_*E*_ is calculated to be 4.7 joules of meat energy per joule of plant energy, which is the average of loss-adjusted *FCR*_*E*_ for beef+lamb, pork, and poultry, weighted by their relative availability in American's diets [43]. Mass-basis feed conversion ratios are reported by Rosegrant et al. [44] as 7, 5, and 2 for beef, pork, and chicken. These values were divided by total proportional loss from primary weight to consumer weight [43] to adjust for processing and waste loss and converted to energy units using their average energy content from [43].

*E*_*PE*_ for dogs and cats is calculated as 453 ± 105 PJ yr^-1^ compared to humans' 1810 ± 16 PJ yr^-1^, resulting in the conclusion that pets' share of the total plant-equivalent energy consumed by pets and humans is 20% ± 6%. Because these calculations involve a ratio with FCR_E_ in both the numerator and the denominator, it is not very sensitive to the actual value of FCR_E_; within a realistic range of estimates for FCR_E_ (2–10), the range of pets' total share of the plant-equivalent energy is 18%–22%. 139 ± 34 million people, eating 8900 kJ d^-1^ (2143 kcal d^-1^) could be supported by the plant-equivalent energy of US dogs and cats, whereas 553 ± 14 million people could be supported by the plant energy equivalent consumed by people in the US.

### Feces production

I used data from Lampe et al. [45] to estimate the average fecal matter produced by people as 0.147 kg capita^-1^ d^-1^, wet weight. De-Oliveira et al. [46] estimate that cats produce 0.042 kg cat^-1^ d^-1^ of fecal matter. Meyer et al. [32] has produced estimates of the amount of fecal dry matter (FDM) and fecal water excretion (FEW) produced by different breeds of dogs with both dry and canned diets. These data were fit to separate power laws (dry: FDM = 46.2 BW^-0.052^, FWE = 2.57 BW^0.059^; canned: FDM = 34.5 BW^-0.111^, FWE = 4.69 BW^0.110^) and values for a dog of estimated mass 22 ± 1.2 kg (calculated above) were calculated. Results for dry and canned diets were averaged and total fecal matter production was calculated as 0.15 ± 0.07 kg dog^-1^ d^-1^. Therefore, the amount of fecal matter (wet weight) produced by dogs, cats, and people in the US is4.4 ± 1.8, 0.72 ± 0.03, and 17 ± 1.3 Tg yr^-1^, respectively. In total, US dogs and cats produce 5.1 ± 1.9 Tg yr^-1^ of feces, which is 30% ± 13% that produced by humans and 23% ± 12% of the total.

Assuming that Americans throw away about 2 kg d^-1^ as garbage [47], if all of the feces from US dogs and cats, not including kitty litter and bags, were disposed as garbage, their feces would be equivalent to the total garbage produced by 6.63 million Americans, or approximately the population of Massachusetts (population 6.64 million in 2015 [48]).

### Relative environmental impact

I followed Reijnders and Soret [6] in determining the environmental impact of dogs’ and cats’ land animal meat consumption. Reijnders and Soret [6] used life cycle analysis to determine the relative impact of producing meat protein compared to producing plant (soy) protein in several categories. In separate calculations for pets and humans, the impact of animal production compared to plant production was calculated as:

$$I_{j}=\frac{\left(1-F_{A}\right){\text{E}}_{C}^{a}+W_{j}F_{A}{\text{E}}_{C}^{a}}{{\text{E}}_{C}^{a}}=\left(1-F_{A}\right)+W_{j}F_{A},$$ (8)

where *I*_*j*_ is the impact of animal production in category *j* (land use, water use, fossil fuels, phosphates, biocides) and *W*_*j*_ is the relative impact of meat protein production (Table 6). The non-animal product energy consumed (i.e., $\left(1-F_{A}\right){\text{E}}_{C}^{a}$) was given an implicit value of unity. The resulting values can be used to determine the relative impacts of pets’ and people’s diets in a way that accounts for varying energetic needs of pets and people.

**Table 6:** Weighting factors for categories of environmental impact.

Category	Relative impact of meat protein production ^a^	Relative impact of plant protein production	(Dog + Cat)/Human
Land Use	6–17	1	26%–29%
Water	4–26	1	25%–30%
Fossil Fuel	6–20	1	26%–30%
Phosphate	7	1	27% ± 5% ^b^
Biocides	6	1	26% ± 5% ^b^

^a^ from Reijnders and Soret [6].

^b^ [6] does not report a range in impacts for these variables, variability is calculated using uncertainty in dog and cat meat/energy consumption

With regard to land use, water, and fossil fuel, the environmental impact of animal production (compared to a plant-protein substitute) used to feed dogs and cats is 25–30% of that used to feed humans (Table 6). For phosphate and biocide use, this proportion is 26–27% ± 5%. An important caveat in these calculations is that the animal-derived energy used includes fish. The approach of Reijnders and Soret [6] is strictly for land animals, which have clear land use, water, fossil fuel, phosphate, and biocide impacts. There is no clear way to determine the amount of fish-derived energy as a proportion of total animal-derived energy in animal feed. However, if the number is similar to that in food consumed by people in the US (~1%, [28]), then the proportional calculations are approximately correct. An additional caveat in the interpretation of these calculations is that they do not differentiate between different sources of animal protein, which can have distinctly different environmental footprints. We used the range/uncertainty provided by Reijnders and Soret and Pimentel and Pimentel [6, 7] to provide reasonable bounds on these results. Without market-wide knowledge of recipe and sales data, much of which is proprietary, a more detailed calculation is not possible. However, this does not mean that these calculations are not valuable to provide an estimate of the scale of the contribution of dogs and cats to these environmental impacts.

### Non-CO_2_ greenhouse gas production

Eshel and Martin [41] calculated ~0.8 kg cap^-1^ yr^-1^ CO_2_-eq due to livestock-related non-CO_2_ greenhouse gas (GHG) emissions (specifically methane and nitrous oxide) produced nationwide. Assuming the mean American diet (with inefficiencies) and multiplying by the population of the US yields an estimate of 260 million ton yr^-1^ CO_2_-eq methane and nitrous oxide produced in the course of livestock production in the US. The proportion of total animal-derived energy consumed by dogs and cats is 25% ± 6% and thus the pets' share of the livestock-related methane and nitrous oxide production is up to 64 ± 16 million tons CO_2_-eq GHG, although this number may be lower because, presumably, there is less waste in the production of dog and cat food.

## Discussion

People love their pets [49]. They provide a host of real and perceived benefits to people including companionship [50], increased physical activity [51], improved mental health and social capital [52], benefits for child development [53], and social status [54]. Many dogs are also working dogs and have roles in assisting the disabled, contributing to military and civilian security, and in traditional roles on ranches and farms. Cats, too, have traditional roles in pest control in addition to their roles as pets. This analysis does not mean to imply that dog and cat ownership should be curtailed for environmental reasons, but neither should we view it as an unalloyed good. It is clear that a transition to pets that eat less meat, and therefore have less environmental impact, would reduce the overall US consumption of meat.

The results presented here indicate that exclusion of pets in calculations of food consumption can skew considerably estimates of the total energy actually consumed. As calculated, US dogs and cats consume as much dietary energy as ~62 million Americans, which is approximately one-fifth of the US population. Although there are fewer dogs and cats in the US than people, they derive more of their energy from animal-derived products (33% ± 6% vs. 19% for people). Thus, if pets' consumption was included in calculations, the US would be equivalent to a country of ~ 380 million in terms of raw dietary energy consumed and a country of about 690 million in terms of animal-derived energy consumed. Thus, it is clear, at least for countries with considerable populations of dogs and cats like the US, that the consumption by these animals should be considered when calculating national food consumption. Their smaller size, lower energetic needs, and primarily herbivorous biology means that small pets, like birds, rodents, and reptiles consume less animal-derived energy making them less important in these calculations.

It could be argued that dogs and cats eat meat that humans cannot consume and which is simply a byproduct of production for human use, and therefore should not be counted as consumption beyond that of humans. To some extent, this is certainly true; humans, for instance, do not generally consume bone meal, a common ingredient. But other ingredients in pet food that are byproducts of human meat production are certainly edible after processing. The argument that dogs' and cats' environmental and energetic impacts are obviated by the fact that they eat byproducts from the human food system, and that otherwise the material would go to waste, relies on the assumption that these same byproducts could not be made to be suitable for human consumption after suitable processing. And much pet food probably is already edible and serves as a potential source of protein as a food of last resort; there are reports, both official and unofficial, of impoverished Americans eating pet food as a necessary supplement to their diet [55–57, 58, 59]. At any rate, the trend toward premium pet food with more animal products that Americans would recognize as edible indicates that pets are eating animal products that could also be eaten by humans and that there is direct competition with the human food system for ingredients in some of these products [10].

The proprietary nature of and incredible variety in pet food recipes makes a detailed calculation impossible, but for the sake of argument, if just one-quarter of the estimated 33% animal-derived energy in pet food was consumable by humans, it alone would support the animal-derived energy consumption of 26 million Americans (with 19% of their energy in derived from animal products). This same energy is equal to the entire energy requirement of almost 5 million Americans, or approximately the population of Colorado [48]. If animal-derived energy was converted to its plant equivalent, one-quarter of the animal-derived energy in US dogs' and cats' food would support ~35 million humans. If even only 5% of the animal-derived energy in pet food could be eaten by humans, this would be equivalent to the animal-product consumption of more than 5 million Americans, and the total energy consumption of 1 million Americans, or about the population of Montana [25, 26, 48].

Additional research is needed to evaluate the animal content and human-edibility of ingredients in dog and cat food after processing, but the calculations presented here indicate that these pets comprise a significant proportion of US energy and animal-derived product consumption, with the consequent environmental impacts, including greenhouse gas emission and feces production. Inasmuch as increasing animal production is a threat to the sustainability of the global food system [1, 2], the non-negligible contribution of dogs and cats compounds the problem and exacerbates the threat to sustainability posed by our dietary choices. This is particularly true given increasing pet ownership in some developing countries, and trends in "humanization" of pet food [22, 23] which competes directly with the human food system [10]. Reducing the rate of dog and cat ownership, perhaps in favor of other, less energy-intensive, pets that offer similar health, social, and emotional benefits, would considerably reduce America's overall livestock-related environmental impacts. Both small (e.g., birds, hamsters) and large (e.g., horses) have been shown to be associated with important benefits, including friendship, verbal interaction, companionship [60, 61], promoting self-care [62, 63], and increased empathy [64]. For children, both small and large pets provide friendship, love, and fun as well as opportunities to learn responsibility and deal with pet mortality and mourning [65, 66]. For children with illnesses, small pets have been shown to improve their attitude and help them keep their minds off their disease [67].

It is not just what we feed pets, but how we feed them that contributes to the environmental impacts of our pets, and obesity is a major problem among domestic animals [68, 69]. The pet food industry has also started to confront the issue of the sustainability of feeding pets through advances in product design, manufacturing, education, and policy in order to reduce overfeeding and waste, encourage recycling, and find alternative sources of protein [10]. Simple measures like feeding domestic dogs and cats nutritionally appropriate amounts will certainly reduce their environmental and energetic impact. However, without large-scale reduction in their number and changes to the food system that drastically reduces the per-capita animal product consumption, the environmental and energetic impact of these animals will remain significant.

## Supporting information

S1 Table Products and ingredients used for calculation of the percent of animal-derived energy (Table 5). The ‘Nth Ingredient Equivalents’ are the Table 3 ingredients with known nutritional content that were equated with the ingredients as listed.
(CSV)
